# Editorial: Lipid metabolism dysregulation in obesity-related diseases and neurodegeneration

**DOI:** 10.3389/fendo.2025.1564003

**Published:** 2025-02-11

**Authors:** Jialiu Zeng, Chih Hung Lo

**Affiliations:** ^1^ Department of Biomedical and Chemical Engineering, Syracuse University, Syracuse, NY, United States; ^2^ Interdisciplinary Neuroscience Program, Syracuse University, Syracuse, NY, United States; ^3^ Department of Biology, Syracuse University, Syracuse, NY, United States

**Keywords:** obesity, neurodegenerative diseases, body-brain interactions, metabolic dysfunction, neuroinflammation, therapeutic targets, biomarker discovery

## Introduction

**Figure 1:**
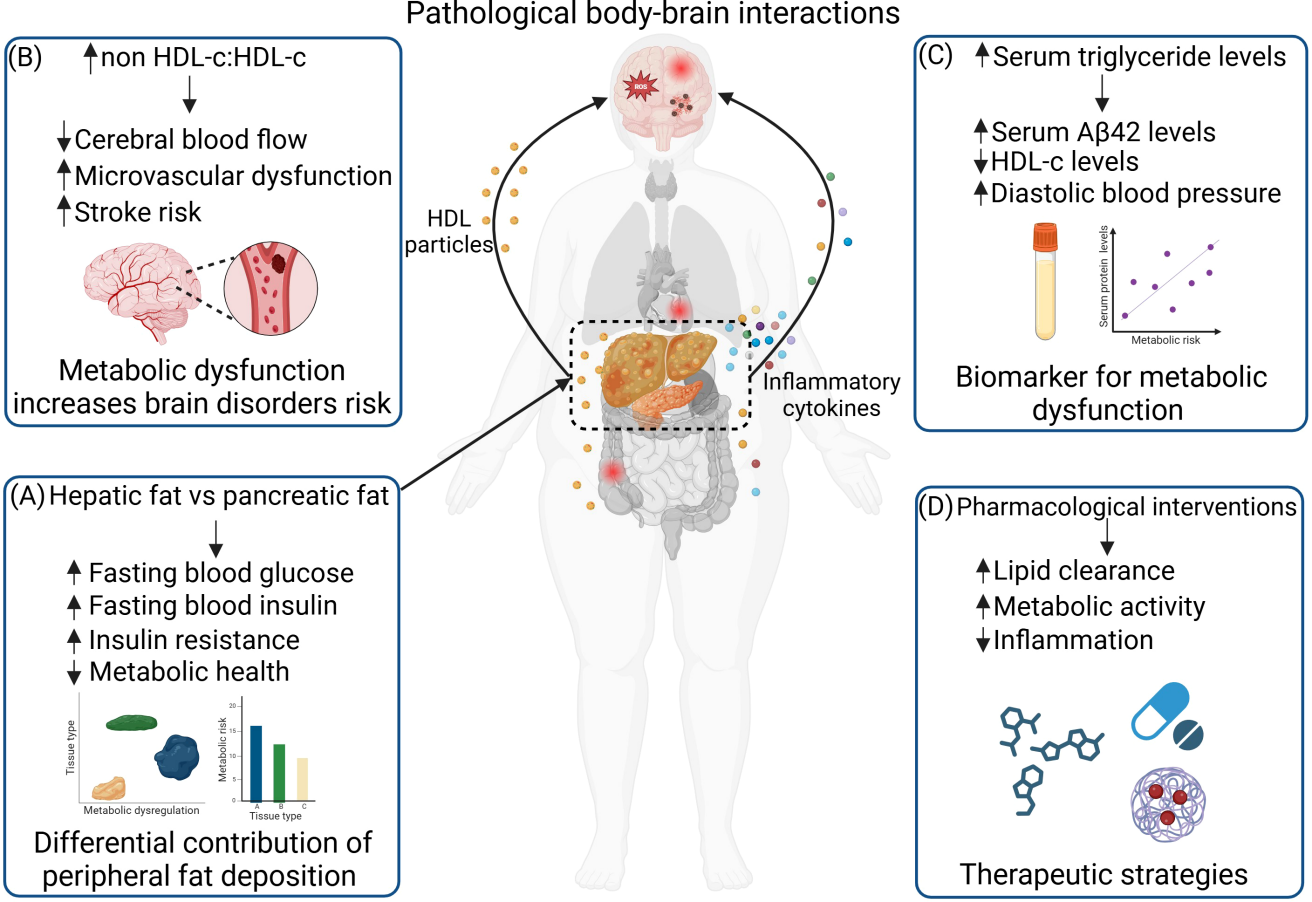
Peripheral metabolic dysfunction drives brain disorders through pathological body-brain interactions. Peripheral fat accumulation in organs such as the liver and pancreas leads to metabolic dysfunction, accompanied by the release of high-density lipoprotein (HDL) cholesterol particles and inflammatory cytokines. These changes not only affect other peripheral organs, such as the gut and cardiovascular system, but also propagate to the brain, contributing to neuroinflammation and neurodegeneration. **(A)** Differential contributions of peripheral fat deposition: Hepatic fat plays a more significant role than pancreatic fat in metabolic dysfunction. Hepatic fat accumulation is associated with increased fasting blood glucose, elevated insulin levels, and greater insulin resistance. **(B)** Metabolic dysfunction elevates the risk of brain disorders: An increased non high density lipoprotein cholesterol (non-HDL-c) to HDL-c ratio negatively impacts cerebral blood flow, exacerbates microvascular dysfunction, and increases stroke risk. **(C)** Biomarkers of metabolic dysfunction: Serum Aβ42 levels positively correlate with serum triglyceride levels and diastolic blood pressure, while negatively correlating with HDL-c levels. **(D)** Therapeutic strategies: Pharmacological interventions can be applied to target metabolic dysfunction and its associated impact on the brain through increasing lipid clearance, increasing metabolic activity, and reducing inflammation. Created in BioRender.

Excessive lipid accumulation disrupts systemic homeostasis, contributing to conditions such as metabolic-associated steatotic liver disease (MASLD), diabetes, cardiovascular diseases, and neurodegenerative disorders (1–3). Lipid metabolism dysregulation, often associated with obesity, plays a pivotal role in metabolic disorders and their intricate interplay with neurological diseases. This body-brain interaction underscores the relationship between peripheral metabolic disturbances and central nervous system dysfunction (4, 5). As ectopic fat deposition, particularly in the liver, heightens systemic inflammation and metabolic stress, it also exacerbates neuroinflammatory pathways and intrinsically disordered protein aggregation (6–12), linking metabolic syndrome to conditions like Alzheimer’s disease (AD), Parkinson’s disease, and vascular dementia (**Figure 1**). Understanding these mechanisms provides a foundation for identifying potential prognostic markers or biomarkers and developing targeted therapeutic strategies that address the shared pathological processes of metabolic and neurological diseases, emphasizing the importance of holistic approaches to health management. This Research Topic highlights important insights into the heterogeneity of fat deposition in different organ tissues under obesity conditions, the association of high-density lipoprotein cholesterol (HDL-c) and non-HDL-c with risks of metabolic and neurological disorders, as well as providing translational insights into potential biomarkers and therapeutic targeting for obesity-related metabolic and neurological disorders that might be linked through body-brain interaction.

## Heterogeneity in peripheral fat deposition and the association with metabolic diseases

Deng et al. highlights the differential contributions of hepatic and pancreatic fat deposits to metabolic risks. This study underscores that liver fat plays a more significant role in influencing metabolic health compared to pancreatic fat. In obesity, ectopic fat accumulation disrupts normal organ functions, and the liver emerges as a dominant factor. Hepatic fat impacts glucose metabolism, lipid profiles, and systemic inflammation, contributing to conditions like insulin resistance and diabetes (**Figure 1A**). He et al. identifies the alanine aminotransferase (ALT) to HDL-c ratio as a potential indicator for diabetes risk in the Chinese population. A high ALT/HDL-c ratio correlates positively with the risk of diabetes development. This ratio reflects a significant association between liver function and metabolic health, as ALT levels are indicative of liver inflammation. ALT behaves similarly to non-HDL-c, which is a harmful lipid marker, highlighting the liver’s role as a major contributor to metabolic dysfunction. These studies emphasize the necessity of tailored strategies to mitigate liver-related metabolic risks, particularly in obesity management, where targeting liver fat reduction and addressing hepatic inflammation might yield more significant health benefits in metabolic disease interventions.

## Connection between metabolic dysfunction and brain disorders

Hernandez Torres et al. examines the link between hyperlipidemia and increased microvascular dysfunction in mice, leading to reduced cerebral blood flow and impaired remote memory, which are key indicators of vascular dementia (**Figure 1B**). A Western diet high in cholesterol exacerbates heart-related issues, which cascade into brain complications. This research emphasizes the detrimental effects of non-HDL-c, a marker of cardiovascular risk, on brain health. Wang et al. explores the complex relationship between the non-HDL-c to HDL-c ratio and stroke risk in middle-aged and older adults (**Figure 1B**). This prospective cohort study demonstrates that a high non-HDL-c/HDL-c ratio is associated with increased stroke risk, particularly in the context of obesity, diabetes, and other co-variates. Interestingly, the study also identifies a non-linear relationship, indicating that stroke risk plateaus once the non-HDL-c/HDL-c ratio surpasses a certain threshold. These studies suggest that maintaining a balanced cholesterol profile is essential for vascular function, underscoring the need for early cholesterol management to preserve cardiovascular and brain health.

## Translational strategies targeting the body-brain interaction

Li et al. investigates the correlation between serum amyloid beta 42 (Aβ42) levels with metabolic syndrome components, including obesity (**Figure 1C**). Their findings indicate that serum Aβ42 levels were positively associated with metabolic syndromes and related clinical parameters, such as body mass index and diastolic blood pressure, while showing a negative association with HDL-c levels in the Chinese population. While Aβ42 is traditionally linked to AD, this study shows that higher serum triglyceride levels contribute to elevated serum Aβ42, which could serve as a biomarker for metabolic syndromes rather than AD. Zeng et al. focuses on therapeutics that address peripheral inflammation and metabolic dysfunction as well as neuroinflammation and neurodegeneration driven by obesity and metabolic disorders (**Figure 1D**). This study presents a comprehensive approach to addressing the complex body-brain interaction by employing diverse therapeutic strategies. These include interventions to modify gut dysbiosis, lifestyle adjustments, dietary supplementation, and the use of pharmacological agents derived from natural sources, all aimed at mitigating obesity-induced neuroinflammation and neurodegeneration. These strategies prioritize restoring systemic metabolic balance to prevent inflammation that could propagate to neuroinflammation and drive neurodegeneration, highlighting the interplay between metabolic health and cognitive function.

## Body-brain interaction: bridging metabolic and neurological functions

The interplay between metabolic health and brain function is a recurring theme across these studies. Obesity, MASLD, diabetes, and lipid imbalances are central drivers of systemic inflammation, liver dysfunction, and vascular abnormalities, all of which have cascading effects on brain health. From the role of heterogenous peripheral fat deposition in metabolic risks to targeting the metabolic-inflammatory axis to mediate cognitive disorders, these findings illuminate the multifaceted connections between the body and the brain. Therapeutic interventions could adopt an integrated approach to address these interconnected systems. Managing liver health, controlling lipid profiles, and targeting inflammation are key steps in breaking the vicious cycle of metabolic and neurological decline (**Figure 1D**). Strategies involving the use of small molecules, nanoparticles, and advanced therapeutic modalities that target either the body, brain or body-brain interactions (13–16) hold significant potential to achieve these goals effectively. Integrative bioinformatics approaches could be applied to gain deeper insights into how different organs or tissues contribute to the metabolic-inflammatory axis (17–19). Understanding these changes may also facilitate the identification of biomarkers for metabolic syndromes and neurodegenerative diseases (20–22). This body-brain perspective not only deepens our understanding of disease mechanisms but also opens new avenues for preventative and therapeutic strategies, paving the way for comprehensive solutions to modern health challenges.
